# Circulating tumor cells as liquid biomarker for high HCC recurrence risk after curative liver resection

**DOI:** 10.18632/oncotarget.21208

**Published:** 2017-09-23

**Authors:** Johann von Felden, Kornelius Schulze, Till Krech, Florian Ewald, Björn Nashan, Klaus Pantel, Ansgar W. Lohse, Sabine Riethdorf, Henning Wege

**Affiliations:** ^1^ Department of Internal Medicine, University Medical Center Hamburg-Eppendorf, 20246 Hamburg, Germany; ^2^ Institute of Pathology, University Medical Center Hamburg-Eppendorf, 20246 Hamburg, Germany; ^3^ Department for Hepatobiliary and Transplant Surgery, University Medical Center Hamburg-Eppendorf, 20246 Hamburg, Germany; ^4^ Department of Tumor Biology, University Medical Center Hamburg-Eppendorf, 20246 Hamburg, Germany

**Keywords:** hepatocellular carcinoma, CTC, BCLC A, liquid biopsy, precision medicine

## Abstract

**Background:**

Early hepatocellular carcinoma (HCC) has a limited prognosis due to recurrence rates of more than 50% after liver resection. Recurrence within two years is believed to be caused by untraceable micro metastases at the time of resection. The objective of this study was to investigate EpCAM-positive circulating tumor cells (CTC) as liquid biomarker to identify patients with high risk of recurrence after liver resection.

**Methods:**

61 patients undergoing resection between 2011 and 2015 were consecutively enrolled. Blood specimens were obtained prior to surgery and processed with the CellSearch^TM^ system, detecting EpCAM-positive CTC. The primary endpoint was recurrence-free survival (RFS).

**Results:**

13 women and 44 men (63.6 ± 11.1 years) were finally evaluated. CTC-positive patients had a significantly higher risk of recurrence with a hazard ratio (HR) of 2.3 (p=0.027), and a shorter RFS compared to CTC-negative patients (5.0 ± 1.5 vs. 12.0 ± 2.5 months, p=0.039). As expected, incomplete resection (R1) was also associated with shorter RFS (HR=2.6, p=0.035), but vascular invasion was not. However, the predictive power of CTC status was independent of R1.

**Conclusion:**

Bloodstream detection of CTC prior to curative-intended liver resection discloses an elevated risk of HCC recurrence and could identify patients, who might benefit from adjuvant treatment.

## INTRODUCTION

With approximately 780,000 new cases worldwide in 2012, liver cancer is ranked second as leading cause of cancer-related death and progressively increases in incidence in the Western world [1, 2]. With 90% of cases, hepatocellular carcinoma (HCC) constitutes the most frequent primary liver cancer to date [3]. The principal clinical stratification scheme is the Barcelona Clinic Liver Cancer (BCLC) staging system; and based on this staging system, only a minor proportion of patients is amenable for curative treatment [4–6]. Without liver transplantation, even early HCC (BCLC stage A) has a limited prognosis due to recurrence rates of 50 – 70% after complete surgical resection or ablation [7–11]. In clinical practice, a cut-off of 24 months is established to distinguish between early and late recurrence or, in other words, recurrence due to progressive intrahepatic micro metastases, which are untraceable by current imaging techniques at the time of resection, or *de novo* tumor formation in the cirrhotic liver [7, 12, 13]. Hence, detection of micro metastases or metastases-initiating circulating tumor cells (CTC) might be crucial to improve curation rates in BCLC stage A patients following resection or ablation, in particular by stratifying patients for liver transplantation and/or early adjuvant treatment. To this regard, a sensitive and specific diagnostic tool to detect metastatic disease in early stage HCC (BCLC stage A) is needed to optimize treatment algorithms.

In a recent work, we employed the CellSearch^™^ system (CSS) as a diagnostic tool for CTC detection and demonstrated that the presence of CTC is associated with systemic disease and inferior overall survival [14] in HCC. The CSS, initially introduced by Cristofanilli *et al.*, detects epithelial cell adhesion molecule (EpCAM)-positive CTC in peripheral blood and has demonstrated an impact on risk stratification and disease management in breast cancer and other malignancies [15, 16]. It has been approved by the U.S. Food and Drug Administration (FDA) for the detection of CTC in patients with metastatic breast, colon, and prostate cancer. In HCC, application of the CSS demonstrated a significant association between advanced disease stages, shorter overall survival, and CTC-positivity [14]. These results have suggested a potential prognostic role for CTC-positivity, which has also recently been demonstrated for cholangiocarcinoma in a small cohort of patients [17]. Employing a less standardized approach, Ogle *et al.* introduced an imaging flow cytometry method, using cytokeratin and EpCAM among other markers, and demonstrated a significant association with overall survival [18]. A second study, representative of a Chinese HCC cohort (90% hepatitis B-associated HCC), revealed a predictive power of EpCAM-positive CTC for tumor recurrence after liver resection [19]. In summary, detection of CTC as liquid biomarker may predict the risk of recurrence after resection at least in Asian HCC cohorts and could hold the capacity to guide treatment decisions in patients with early HCC. Hence, the aim of this study was to verify CTC detection in early-staged HCC (BCLC stage A) in a Western cohort of patients, to identify patients with high risk of HCC recurrence.

## RESULTS

### Patient characteristics

**Figure 1:**
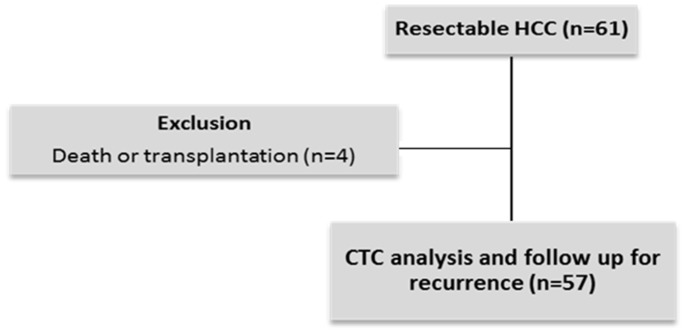
Study design Displayed are the numbers of patients in each stage of the analysis and reasons for exclusion.

Patient baseline characteristics are displayed in Table 1. The final cohort contained 13 (22.8%) female and 44 (77.2%) male patients with a mean age of 63.6 ± 11.1 years at diagnosis, demonstrating a representative Western HCC cohort as described in the literature (study design in Figure 1). Etiologies of the underlying liver disease were assessed from (a) the electronic patient chart, (b) the pathology report of adjacent non-cancerous liver tissue, if available, or (c) both, and were as follows: Chronic viral hepatitis (n=9 chronic hepatitis B, n=1 coinfection with hepatitis B and D, n=9 chronic hepatitis C), chronic alcohol abuse (n=5), a combination of chronic alcohol abuse and hepatitis C (n=2), non-alcoholic steatohepatitis (NASH; n=10), venous occlusive disease (n=2), primary biliary cholangitis with secondary autoimmune hepatitis (n=1), and cryptogenic cirrhosis (n=2). 16 patients had no detectable underlying liver disease. Liver cirrhosis was present in 24 cases (42.1%). Noteworthy, 13 out of the 33 non-cirrhotic patients had NASH (n=8) or chronic hepatitis B (n=5) as underlying liver disease. This reflects standard patient selection for curative resection in Western tertiary referral centers. A high number of patients with liver cirrhosis is not suitable for resection, e.g. due to portal hypertension. On the other hand, resection is the recommended first-line treatment for patients with HCC without underlying liver cirrhosis also for large tumors [7].

**Table 1:** Demographic characteristics of patients with resection of HCC and CTC analysis

Baseline Characteristics	Patients, n=57 (%)
**Gender (%)** Male Female	44 (77.2) 13 (22.8)
**Age (years)** Mean ± SD Minimum Maximum	63.6 ± 11.1 33 84
**Liver cirrhosis (%)** Yes No	24 (42.1) 33 (57.9)
**Child-Pugh status (%)** A B	18 (75.0) 6 (25.0)
**Etiology of liver disease** Alcohol^#^ Chronic viral hepatitis^#^ NASH Venous occlusive disease PBC with secondary AIH Cryptogenic cirrhosis None	7 21 10 2 1 2 16
BCLC staging^*^ A B	22 2
**Tumor Grading (G)^§^** 1 2 3	15 38 3
**Tumor size (T)^§^** 1 2 3	26 18 12
**Vascular invasion (V)^§^** 0 1 2	35 20 1
**Resection margin (R)^§^** 0 1 2	49 7 0
**Recurrence within 2 years (%)** Yes No	36 (63.2) 21 (36.8)

Abbreviations: SD, standard deviation; NASH, non-alcoholic steatohepatitis; PBC, primary biliary cholangitis; AIH, autoimmune hepatitis; T, tumor size; V, vascular invasion; R, resection margin. ^#^Two patients revealed multiple risk factors. ^*^Only applicable for cirrhotic patients. ^§^In one patient histopathological analysis was not possible due to complete necrosis after prior treatment.

### CTC analysis

Of all patients, nine cases were tested positive for CTC. Five of them revealed one CTC, two cases two CTC, and another two cases were positive with three CTC. As described before [14, 15], CTC detection applying the CSS is highly sensitive and specific. Therefore, unbiased CTC calls before resection in this study are reliable, even considering the low number of CTC detected. In the remaining 48 patients, no CTC were detected. The baseline characteristics of CTC-negative and CTC-positive patients are displayed in Table 2. Regarding gender, age, and presence of underlying chronic liver disease, patients were equally distributed between the two groups. Of note, all nine patients, who were tested CTC-positive, suffered from virus-related HCC (n=5 with chronic hepatitis B, with one of them being co-infected with hepatitis D virus; n=4 with chronic hepatitis C, including one patient with chronic alcohol abuse as an additional risk factor). This surprising and currently unexplained finding resulted in a significant distribution of virus-related HCC between CTC-positive and CTC-negative cases (Pearson's Chi Square p=0.006). Histopathological parameters like tumor size (T status), grading, vascular invasion (V status), and positive resection margin (R status) were equally distributed between the two groups.

**Table 2:** Baseline characteristics of CTC-negative and CTC-positive patients

	Patients		
	CTC-negative, n=48 (%)	CTC-positive, n=9 (%)	P-value
**Gender (%)** Male Female	36 (75.0) 12 (25.0)	8 (88.9) 1 (11.1)	0.362
**Age (years)** Mean ± SD	64.2 ± 12.0	61.4 ± 4.2	0.528
**Liver cirrhosis (%)** Yes No	18 (37.5) 30 (62.5)	6 (66.7) 3 (33.3)	0.104
**Child-Pugh classification (%)** A B	13 (75.0) 5 (25.0)	5 (83.3) 1 (16.7)	0.950
**Etiology of liver disease** Alcohol^#^ Chronic viral hepatitis^#^ NASH Venous occlusive disease PBC with secondary AIH Cryptogenic cirrhosis None	6 12 10 2 1 2 16	1 9 0 0 0 0 0	0.006
BCLC staging^*^ A B	17 1	5 1	0.446
**Tumor Grading (G)^§^** 1 2 3	13 32 2	2 6 1	0.403
**Tumor size (T)^§^** 1 2 3	23 13 1	3 5 1	0.410
**Vascular Invasion (V)^§^** 0 1 2	30 16 1	5 4 0	0.639
**Resection margin (R)^§^** 0 1	41 6	8 1	0.891
**Recurrence** N (%) RFS in months	28 (58) 12.0 ± 2.5	8 (89) 5.0 ± 1.5	0.027

Abbreviations: SD, standard deviation; NASH, non-alcoholic steatohepatitis; PBC, primary biliary cholangitis; AIH, autoimmune hepatitis; T, tumor size; V, vascular invasion; R, resection margin. ^#^Two patients revealed multiple risk factors. ^*^Only applicable for cirrhotic patients. ^§^In one patient histopathological analysis was not possible due to complete necrosis after prior treatment.

### HCC recurrence

**Figure 2:**
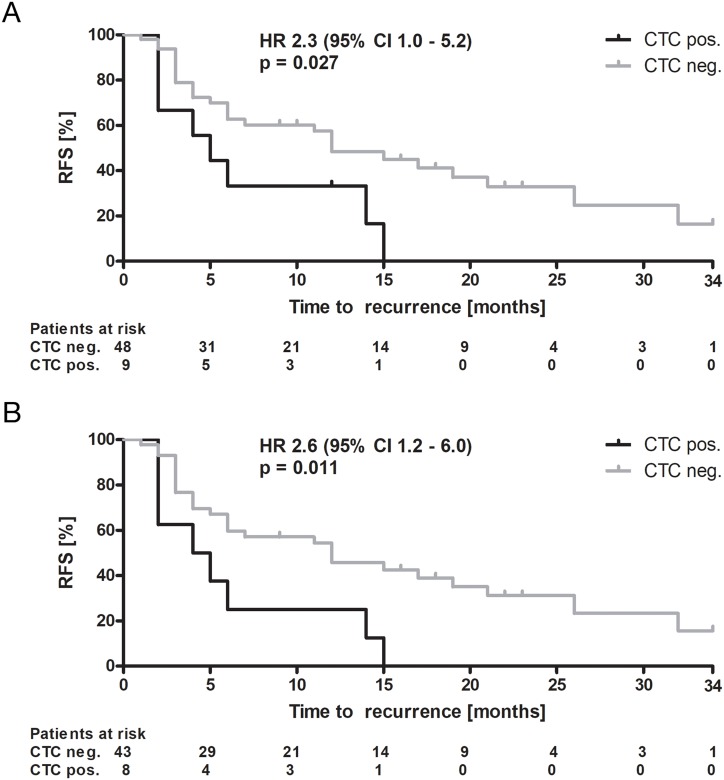
Recurrence-free survival Kaplan-Meier curves of **(A)** the overall cohort and **(B)** a sub-cohort excluding patients who died during the observation period without confirmed data regarding status of recurrence. Displayed are recurrence-free survival (RFS) rates over time according to CTC status (negative vs. positive), hazard ratio (HR) with 95% confidence interval (CI) and level of significance according to log-rank (Mantel-Cox) test. Abbreviations: CTC, circulating tumor cells; neg., negative; pos., positive.

36 patients in our cohort of 57 patients (63.2%) were diagnosed with recurrent HCC within two years with a median time to recurrence of 5.0 ± 7.5 months. Employing a Cox regression analysis, CTC-positive patients had a hazard ratio (HR) of 2.3 for recurrence of HCC compared to CTC-negative patients (95% CI 1.0 – 5.2, n=57, log rank Mantel-Cox p=0.027, see Table 3). The RFS for CTC-positive patients was significantly shorter with a median of 5.0 ± 1.5 months compared to CTC-negative patients with a median of 12.0 ± 2.5 months (p=0.027) (see Figure 2A).

**Table 3:** Univariate and multivariate Cox regression analysis regarding recurrence of HCC

	Univariate HR (95% CI)	P-value	Multivariate HR (95% CI)	P-value
**CTC status**	2.3 (1.0-5.2)	0.027	3.1 (1.0-9.4)	0.043
**Tumor status**	1.3 (0.9-1.9)	0.226		
**Grading**	2.6 (0.9-8.9)	0.107		
**Vascular invasion**	0.8 (0.4-1.7)	0.634		
**Resection margin**	2.6 (1.1-6.4)	0.035	3.7 (1.4-10.3)	0.011
**Liver cirrhosis**	1.1 (0.6-2.2)	0.754		
**Viral etiology**	1.3 (0.6-2.8)	0.438		

Cox regression analysis on the risk of HCC recurrence after resection, n=57. Abbreviations: HR, hazard ratio; CI, confidence interval.

Of the overall cohort, five patients died during follow-up without confirmed data regarding recurrence (all within 2 – 12 months after resection), one of them being CTC-positive. Another patient was allocated for liver transplantation ten months after resection and died one month later due to complications of transplantation with arterial thrombosis and unsuccessful re-transplantation. To strengthen our results and to avoid misinterpretation, we performed a sub analysis excluding these 6 patients without definite knowledge regarding their recurrence status, leaving 51 patients. Despite the lower number of patients included, the results still revealed a significantly higher risk for recurrence of HCC in CTC-positive patients (HR 2.6, 95% CI 1.2 – 6.0, n=51, log rank Mantel-Cox p=0.011, see Figure 2B).

Noteworthy, eight of nine and eight of eight CTC-positive patients had HCC recurrence in the overall cohort and sub cohort, respectively, resulting in a positive predictive value of 89% and 100%, respectively. On the other hand, only 28 out of 48 CTC-negative patients (58%) suffered from recurrence of HCC.

As expected, the presence of a positive resection margin (R1 status) was also significantly correlated with recurrence of HCC with a HR of 2.6 (95% CI 1.1 – 6.4, n=57, p=0.035, see Table 3). However, even in this sub-cohort of R1-patients, median RFS for CTC-positive patients was significantly shorter with a median of 4.0 ± 0.8 months compared to CTC-negative patients with a median of 12.0 ± 1.7 months (data not shown, statistical analysis due to small sample size not performed). Tumor size (T status), grading, vascular invasion (V status), the presence of liver cirrhosis, and viral etiology of the underlying chronic liver disease had no significant influence on the recurrence of HCC after liver resection. A covariate Cox regression confirmed these results. The influence of CTC and R status remained significant and independent including these two variables in multivariate Cox regression (CTC-status: HR 3.1, 95% CI 1.0 – 9.4, n=57, p=0.043; resection status: HR 3.7, 95% CI 1.4 – 10.3, n=57, p=0.011, see Table 3). All other variables were not included in multivariate regression because of lack of significance in univariate analysis.

## DISCUSSION

The aim of this study was to investigate EpCAM-positive CTC detection by employing the semi-automated CSS as a liquid biomarker, and thereby to identify patients with high HCC recurrence risk after curative liver resection. The primary and clinically relevant endpoint was RFS at two years, since early tumor recurrence within two years is believed to be caused by intrahepatic micro metastases at time of resection, which are untraceable by current imaging techniques. By consecutively enrolling and analyzing 57 patients with early-staged HCC (BCLC stage A) in this prospective mono-center study, we demonstrate, based on an unbiased, highly standardized, and reliable method to detect CTC before resection, that preoperative detection of CTC indicates a significantly increased risk of recurrence after liver resection compared to CTC-negative patients with a HR of 2.3 (95%-CI 1.0 – 5.2). The positive predictive value of CTC detection for recurrence of HCC was 89% overall, and 100% in a sub cohort excluding patients for whom the recurrence status remained unclear. Furthermore, RFS for CTC-positive patients was significantly shorter with a median of 5.0 ± 1.5 months compared to 12.0 ± 2.5 months in CTC-negative patients, supporting the paradigm of micro metastases as the key mechanism for early recurrence in patients with early HCC, who undergo potentially curative treatment concepts. In our cohort, only two patients suffered from recurrence >24 months after resection (26 and 32 months after resection, respectively), both within the group of CTC-negative patients, whereas all CTC-positive patients recurred within 15 months after resection. Considering the paradigm, that recurrence of HCC >24 months after resection might as well display *de novo* HCC in a precancerous liver (both patients had cirrhosis), a sub analysis based only on recurrence within 24 months after resection showed statistically robust results without any impact on the statistical values (data not shown).

Therefore, CTC detection could potentially identify HCC patients with occult metastatic disease. Our study raises the question for prospective follow-up studies to investigate if CTC-positive patients undergoing resection (or possibly even ablation) would benefit from neoadjuvant or adjuvant treatment options.

To date, systemic treatment options are limited in patients with HCC [20–23]. In fact, only the two multi-targeted tyrosine-kinase inhibitors Sorafenib and Regorafenib are effective in randomized controlled trials as first- and second-line therapy in advanced disease stages. Adjuvant treatment in patients with HCC is a pivotal issue. In line with several other trials, Bruix *et al.* reported that Sorafenib is ineffective with respect to RFS compared to placebo in an adjuvant setting following HCC resection or ablation [24]. Therefore, current guidelines do not recommend adjuvant systemic treatment. However, in the performed trials, patients were not selected by biomarkers predicting their potential risk of recurrence. Uncovering of a systemic or metastatic disease stage by detecting micro metastases or CTC at the earliest time point possible, could be very valuable to improve curative treatment outcome and to select patients with the highest HCC recurrence risk for adjuvant regimens. Future clinical trials should implement this knowledge in selecting patients for adjuvant therapy.

In a recent work by our study group, we have demonstrated that the presence of EpCAM-positive CTC is associated with systemic disease and shortened overall survival [14]. Based on these data, a further study is needed to define prognostic and therapeutic implications of EpCAM-positive CTC detection in patients staged BCLC A or B. In this study, focusing on patients with early stage HCC (BCLC stage A or patients without cirrhosis), we applied the CSS to uncover an occult metastatic disease. As expected, only few patients were tested positive for CTC in our cohort of Western HCC patients. However, CTC-positive patients displayed a significantly shortened RFS. Comparing established risk factors for recurrence, as anticipated, incomplete resection (R1) was identified as an additional parameter associated with shorter RFS. However, the predictive power of CTC status was independent of R1. Microscopic vascular invasion (V1) was equally distributed between patients with and without CTC and showed no correlation with RFS. One should keep in mind that this could be due to technical reasons, since the detection of microvascular invasion in large tumor nodules is challenging. Nevertheless, CTC detection displays a more feasible and statistically independent biomarker compared to microvascular invasion in terms of RFS and thus potentially regarding the existence of micro metastases within the liver. The statistically significant results of CTC detection and the impact of incomplete resection for the prediction of recurrence show that our study is sufficiently powered to already draw meaningful and clinically relevant conclusions.

EpCAM-positive CTC detection in patients with HCC has been assessed by different study groups, implementing diverse diagnostic techniques. Recently, Ogle *et al.* introduced an imaging flow cytometry method, using cytokeratin and EpCAM amongst other markers, and demonstrated a significant association with tumor size, portal vein thrombosis, and ultimately with overall survival in a heterogenic patient cohort, including mainly patients with systemic HCC in BCLC stage C (70%) [18]. Only 14 of the enrolled patients underwent curative treatment. Therefore, in this study it was not possible to evaluate the prognostic significance of CTC detection in early stage HCC (BCLC stage A). Another study group highlighted the clinical relevance of EpCAM mRNA-positive CTC, implementing a qRT-PCR-based platform [25]. This platform showed only a 76.7% consistency with the CSS. To this regard, the CSS offers a high degree of standardization and reproducibility and is approved by the FDA for diverse cancer entities. Two Chinese studies have investigated the predictive power of EpCAM-positive CTC for tumor recurrence after liver resection, and revealed a significant association with early tumor recurrence after liver resection [19, 26]. However, the collected data were obtained from HCC patients suffering from chronic hepatitis B in the vast majority, 100% and >90% of the total cohort, respectively. Therefore, the data is representative for a Chinese patient cohort and lacks sufficient translation to a Western population, in which chronic hepatitis C and alcohol consumption are more frequent risk factors. Moreover, the later study by Zhou *et al.* did not utilize the CSS, but rather an EpCAM mRNA-positive CTC detection method [26].

EpCAM is a preferred biomarker for CTC detection, since this protein is frequently expressed in prognosis-relevant CTC, e.g. in breast cancer [27, 28]. The detection of CTC for prediction of recurrence after resection was also investigated in other tumor entities, e.g. esophageal cancer [29]. Additionally, Yamashita *et al.* identified a subset of cancer cells with stem cell characteristics, e.g. over-expression of EpCAM, within HCC nodules and demonstrated a significant association with enhanced tumor progression, angioinvasion, and overall survival [30]. Hence, although EpCAM over-expression is not a common phenomenon in HCC nodules, it is, based on experimental and clinical data, of foremost relevance for the initiation of metastases and tumor aggressiveness.

The presented study reveals obvious strengths and shortcomings by nature of the study design. Limitations are the mono-center setting and the number of enrolled patients. However, to our knowledge, this is the largest investigation to date in a Western population, screening these rare patients for EpCAM-positive CTC by employing the CSS in a prospective manner over a period of five years. Additionally, the statistical significant results reveal that the cohort size is sufficiently powered. In our opinion, enlargement of the cohort is therefore not necessary and does not add any benefit to our study. The study design did not include a control arm, e.g. patients suffering solitarily from liver cirrhosis, since we and other groups have demonstrated specificity of the CSS in previous studies [14, 19]. As a major advantage, CTC detection in the context of HCC staging is appealing and feasible, because appropriate blood samples are easy to obtain and the intervention is cost-effective, lacks serious side effects, and is quickly performed in daily routine.

Conclusively, bloodstream detection of EpCAM-positive CTC prior to surgery predicts an elevated HCC recurrence risk and shorter RFS after curative resection, independent of vascular invasion (V1) or positive resection margins (R1). EpCAM-positive CTC might serve as a biomarker for metastatic disease, and thus represent the urgently needed marker to identify patients with high requirement for adjuvant treatment. Besides, this is the first study demonstrating a clinical impact of EpCAM-positive CTC detection as liquid biomarker in Western patients with early HCC undergoing curative resection.

## MATERIALS AND METHODS

### Patients

For this study, 61 patients diagnosed with early stage HCC, who underwent liver resection were consecutively enrolled and prospectively followed up between July 2011 and June 2016 at the University Medical Center Hamburg-Eppendorf (see Figure 1). Only patients with histologically proven HCC were included. Recurrence was diagnosed applying current imaging guidelines (mRECIST) as proposed by the European Association for the Study of the Liver (EASL) [7] and confirmation by biopsy in case of inconclusive contrast dynamics. From the initial 61 patients, four patients had to be excluded because of death or liver transplantation within 30 days after resection (see Figure 1). Hence, the final analysis was performed on 57 patients. Patients were acquired from the I. Department of Medicine and the Department of Hepatobiliary and Transplant Surgery, University Medical Center Hamburg-Eppendorf. Exclusion criteria were age <18 years, and active or preexisting other malignancies. The study was approved by the Ethics Committee of the Hamburg Medical Association (approval number PV3578) and written informed consent to the study protocol was obtained from all participants prior to inclusion in this study. Patients were not limited to any type or line of therapy.

### Clinical information

Clinical characteristics such as demographic data, risk factors, underlying chronic liver disease or cirrhosis, tumor stage according to the BCLC staging system [3] and tumor/node/metastasis (TNM) classification (American Joint Committee on Cancer Staging Manual, 7^th^ edition, 2010), and presence of macroscopic or microscopic vascular invasion were recorded at time of liver resection. Data was obtained by review of medical records, including preoperative imaging, and surgical and pathological reports following resection. After liver resection, surveillance of patients was performed with regular cross-sectional imaging of the liver, clinical examination, and laboratory testing every three months according to clinical guideline recommendations. All patients were followed up until recurrence of HCC or effective time of data analysis in June 2016.

### Patient blood samples

Blood specimens (7.5 ml) were drawn 24 hours before surgical treatment into CellSave^™^ Preservative Tubes (Veridex), stored at room temperature and processed within 96 hours after collection. To avoid possible contamination with epithelial cells of the skin, one extra tube was filled prior to the assay tube.

### CellSearch^™^ system (CSS)

The CSS is a semi-automated device detecting and enumerating EpCAM-positive/keratin-positive CTC. First, the automated Celltracks^™^ AutoPrep system enriches cells with ferrofluid-coated anti-EpCAM-antibodies. Next, these cells are immunostained with fluorescently-labeled anti-keratin-antibodies identifying, among others, keratins (CK) 8, 18, and 19. Nuclear staining with 4’,6-diamidino-2-phenylindole (DAPI) ensures integrity of nuclei, and anti-CD45-antibodies distinguish epithelial cells from leukocytes. Afterwards, the CellTracks Analyzer identifies keratin/DAPI-positive images by semi-automated fluorescence-based microscopy for a blinded and experienced observer on a computer desktop. Per definition, identified cells qualify for EpCAM-positive CTC if they were oval or round, contain a nucleus (DAPI staining), express keratin and are CD45-negative. The CSS offers a high degree of standardization and reproducibility and is approved by the FDA for diverse cancer entities.

### Histopathological assessment

The samples were directly obtained during the surgical procedure and the histopathological diagnosis of HCC was confirmed by an expert liver pathologist. Formalin-fixed paraffin-embedded (FFPE) sections of the resected tissue were stained with Hematein-Eosin (HE), and a representative paraffin block from each specimen was chosen for immunohistochemistry analysis. After deparaffinization and rehydratation, antigen recovery was ensured by citrate buffer. Next, sections were incubated with a primary anti-glypican 3 (GPC3) antibody (1/100, clone IG12; Biomosaics, USA), anti-heat shock protein 70 (HSP70) antibody (1/250, clone SC24; Santa Cruz Biotechnology, USA), and anti-glutamine synthetase (GS) antibody (1/500, clone MAB302; Chemicon International, USA), an established combination of markers to specifically identify HCC nodules [31]. After incubation with the Envision detection system by Dako (Glostrup, Denmark), and staining with 3-diaminobenzidine (DAB) as chromogen, the slides were counterstained with Mayer's haematoxylin, dehydrated, and cover slipped. Staining of GPC3 and HSP70 was considered positive when more than 5% of the hepatocytes were immunoreactive. Staining of GS was considered positive when more than 50% of hepatocytes were immunoreactive. Additional histological baseline characteristics according to the TNM classification, grading, micro- and macrovascular invasion, as well as the non-tumoral liver tissue state, according to the METAVIR score (F0-1 non-fibrotic, F2-3 fibrotic, and F4 cirrhotic), were assessed.

### Statistical analysis

Data are presented as median or mean ± standard deviation (SD), as indicated. Statistical analysis was performed with Pearson's Chi Square, univariate and multivariate Cox regression, and Kaplan-Meier curves using IBM SPSS Statistics Version 22 (IBM, USA) and GraphPad Prism Version 4 (GraphPad Software Inc., USA). For Cox regression of non-binary coded parameters, e.g. tumor size (T status), grading, and microvascular invasion (V status), a dichotomous fashion was used as follows: T1/2 vs. T3, G1/2 vs. G3, and V0 vs. V1/2. Multivariate Cox regression was only performed with variables showing significant association in univariate analysis. CTC analysis was nonparametric according to the existence of CTC (CTC negative vs. CTC positive). For all statistical analyses p-values below 0.05 were considered significant.

## Abbreviations

AIH: autoimmune hepatitis
BCLC: Barcelona Clinic Liver Cancer
CD45: cluster of differentiation 45, receptor-type tyrosine-proteinphosphatase C
CI: confidence interval
CK: cytokeratin
CSS: CellSearch^™^ system
CTC: circulating tumor cell
DAB: 3-diaminobenzidine
DAPI: 4’,6-diamidino-2-phenylindole
EASL: European Association for the Study of the Liver
EpCAM: epithelial cell adhesion molecule
F: fibrosis
FDA: Food and Drug Administration
FFPE: formalin-fixed paraffin-embedded
G: grading
GPC3: glypican 3
GS: glutamine synthetase
HCC: hepatocellular carcinoma
HE: Hematein-Eosin
HSP70: heat shock protein 70
HR: hazard ratio
METAVIR: Meta-analysis of Histological Data in Viral Hepatitis
mRECIST: modified Response Evaluation Criteria in Solid Tumors
NASH: non-alcoholic steatohepatitis
PBC: primary biliary cholangitis
qRT-PCR: quantitative real-time polymerase chain reaction
R: resection margin
RFS: recurrence-free survival
SD: standard deviation
T: tumor size
TNM: tumor/nodes/metastasis classification
V: vascular invasion
